# What Is Tubercle?

**Published:** 1882-05

**Authors:** 


### WHAT IS TUBERCLE?

Dr. Sidney Coupland, in recent lectures (*British Med. Journal*), gives the following conclusions based upon his studies on the subject:

1. Tuberculosis is an infective disease, to which man and the higher animals are liable.

2. It is characterized, anatomically, by the formation of minute nodules or "granulations," composed of elements like those met with in granulation tissue, the result of simple reparative inflammation.

3. These nodules, or elementary or primary "tubercles," may occur in an isolated manner, or, by their confluence, may form larger or smaller conglomerate masses.

4. The typical structure of each fully formed primary nodule consists in (*a*) a collection of lymphoid round cells, enclosed in a delicate fibrillar meshwork or stroma; (*b*) in an internal zone, more or less evident, of larger nucleated epithelioid cells; and (*c*) a central multi-nucleated or giant cell.

5. These "tubercles" arise apparently in connection with the lymphatic tissue that pervades the body. No region is exempt from them. They may occur in the substance of organs, in the bones and muscles, in serous membranes, as the pia, arachnoid, pleura, pericardium, and peritoneum; in synovial membranes; in mucous membranes (arising in the sub-mucous stratum), as in the mouth, pharynx, larynx, trachea, bronchi, intestines, and genito-urinary tract.

6. Being ill-supplied with blood-vessels, they can only attain a certain size, and then perish. The central cells degenerate first, because they are the farthest removed from the nutrient blood-stream, and mutual pressure, due to their increasing growth, hampers their vital activity. They become fattily degenerated, soft, opaque, caseous, forming "yellow" tubercles, which, when isolated, are larger and manifestly of older formation than the miliary, translucent, gray granules. Where such tubercles are confluent, larger and more irregular caseous masses are formed. Caseation may pass into cretification. On the other hand, there is no doubt that occasionally the tubercular nodules take on a fibroid change, passing from the stage of "granulation tissue" to one resembling "cicatricial tissue."

7. Almost invariably there occurs, in the vicinity of the tubercular formation, some reactive inflammation. This may be protective by ultimately leading to encapsulation by fibrous tissue, of the caseated tubercular focus ; or, as more frequently happens, it aids in the disintegration of the surrounding tissues, and leads, with the necrosis of the tubercles themselves, to destructive ulceration.

8. Individuals who are prone to the development of tubercle are called "tuberculous." The disposition may be inherited. Probably, what we recognize as "struma" or "scrofula" is only one form of this—a tendency to tuberculosis of lymphatic glands especially ; just as in phthisical subjects we have a tendency to pulmonary tuberculosis.

9. The tubercular manifestation is, in the majority of cases, at first local, *i.e.*, limited to one organ or tissue. It may remain so limited throughout life—may not even endanger life—or may lead to death by the local destruction to which it gives rise. On the other hand, it may be more or less widely diffused throughout the body of the same individual. This diffusion may be due, sometimes, to the simultaneous development of tuberculosis in many parts. More frequently it is due to secondary dissemination by a process of infection.

10. This dissemination takes place, as in cancer, in two ways, viz.: by direct extension, or infection of neighboring tissues by contiguity; and by general distribution of the tubercular virus through the medium of the blood-system (including lymphatics).

11. The tubercular virus seems to be most potent, or, at any rate, to retain its potency, *i.e.*, its infective property, in the caseous state.

12. Examples of the local extension of tubercle, or of propagation by contiguous infection, are seen: (1) in the development of peritoneal tubercle from intestinal;* (2) in the spreading of tubercle from one part of an organ (*e.g.*, lung) to another part; (3) in extension from lung to pleura;* (4) in bronchial, laryngeal, and intestinal ulceration, excited by the passage over their mucous membrane of material expectorated from a phthisical lung; (5) in tuberculosis of bladder and vesiculæ seminales, following upon renal or testicular tubercle, etc. The mode of its local extension approximates tubercle to the neoplasmata, viz., by its elements exciting, in the tissue they infect, changes leading to the formation of cell-masses resembling the primary focus.

13. The generalization of tubercle is shown in the disease known as acute miliary tuberculosis, which is characterized by an eruption of miliary granulations in diverse organs and tissues. Its mode of occurrence may be (as above) compared to the general dissemination of secondary cancer, or perhaps, with equal truth, to the metastatic suppuration of pyæmia. With few exceptions, it appears to necessitate a primary tubercular focus to give rise to it. It is believed that the infective virus, whatever it be, enters the blood-stream at this local focus, and is thence widely disseminated, the resulting growths being, for the most part, miliary, gray, and translucent—life not, as a rule, being prolonged for a sufficient length of time after the occurrence of the generalization to permit of the growths becoming confluent or caseous. As the membranes of the brain are generally involved in this widespread infection, death occurs early.

14. Lastly, tuberculosis is inoculable. In this respect it resembles pyæmia and differs from the cancers; for there is reason to think that it may be and is communicated from one human being to another, *e.g.*, from husband to wife, and *vice versa;* and that it can be inoculated in animals from man (artificial tubercle). There is, further, a possibility, based on a certain peculiar morphological resemblance of the formations, that bovine tuberculosis is communicable to man.

15. If the foregoing data be true, it follows that tuberculosis is an infective disease, probably due to the presence of a virus, which gives rise to the development of peculiar tissue-formation, capable of localized or general propagation in the body, and characterized mainly by their tendency to early disintegration.

16. Until the nature of the virus is known, it is impossible to formulate data concerning the condition under which the disease arises in subjects free from inherited taint.

* In these cases, probably by extension along lymphatic channels.

